# Elderly Activity Life-Space Envelopes (EASE): Development and Feasibility of a Comprehensive, Integrated Protocol for Life-Space Mobility Research in Population Health

**DOI:** 10.2196/79308

**Published:** 2025-12-19

**Authors:** Yee Sien Ng, Fang Zhao, Lynn Yi-Ching Ho, Laura Tay, Eugene Shum, Aisyah Latib, Silvana Choo, Sharon Chew, Teresa Leong, Yong Hao Pua, Belinda Yuen, Sam Conrad Joyce, Xin Yang, Angelique Chan, Ad Maulod, Yunjie Wong, Donny Soh, Rajesh Krishna Balan, Lian Leng Low, Julian Thumboo, Yew Yoong Ding, Helen Marie Hoenig, Sapphire Lin

**Affiliations:** 1 Centre for Population Health Research and Implementation, SingHealth, 10 Hospital Blvd, Singapore, 168582, Singapore, 1 63266667; 2 Singapore General Hospital, SingHealth, Singapore, Singapore; 3 Sengkang General Hospital, SingHealth, Singapore, Singapore; 4 Duke-NUS Medical School, Singapore, Singapore; 5 Geriatric Education and Research Institute, Singapore, Singapore; 6 Mobile Market Monitor, Boston, MA, United States; 7 Singapore University of Technology and Design, Singapore, Singapore; 8 Changi General Hospital, SingHealth, Singapore, Singapore; 9 Singapore Institute of Technology, Singapore, Singapore; 10 Singapore Management University, Singapore, Singapore; 11 Tan Tock Seng Hospital, National Healthcare Group, Singapore, Singapore; 12 Duke University, Durham, NC, United States

**Keywords:** community mobility, community participation, geospatial analysis, preventative health, travel behavior

## Abstract

**Background:**

Life-space mobility (LSM) refers to the movement of people over time and the areas through which they move to achieve life goals of health, employment, security, and participation for active aging.

**Objective:**

The Elderly Activity Life-Space Envelopes program is a large interdisciplinary mixed methods LSM study in older adults of 50 years and above. The overarching aims were to discover why, where, and when older adults travel and how they get to their destinations.

**Methods:**

In this paper, we focus on the methodology of the main quantitative phase. This community-based study comprised an in-person multidomain geriatric assessment with physical performance measures, followed by a 14-day travel diary. For the multidomain geriatric assessment, we structurally underpinned important population health constructs including the WHO International Classification of Functioning, Disability and Health, Frailty, and Intrinsic Capacity. We also described home meso-environments by incorporating authoritative open-source environmental attributes. These facilitated the categorization of LSM determinants into health, social, and environmental domains. The LSM outcomes include self-reported and objective geographical information science LSM measures. We further developed a suite of geographical information science LSM outcomes in alignment with our overarching aims. Quota sampling based on age groups, housing typology, and frailty status was applied. A customized, smartphone-based digital travel diary was designed, and barometric sensors were enabled to capture 3D LSM in capable smartphones.

**Results:**

We recruited 1131 older adults with an average age of 63.8 (SD 7.6) years. The large majority (n=1062, 95%) successfully documented their travel diary on their smartphones with the rest on paper-based travel diaries. For the digital data, a total of 88,166 node points were recorded. There were 76,741 trips and 106,323 trip legs documented through the e-travel diary platform. Valid vertical LSM data were obtained from 228 participants. The majority (n=842, 75%) lived in public apartments, and 29% (n=326) were prefrail or frail.

**Conclusions:**

We provide a practical, feasible yet comprehensive protocol integrating LSM within important population health themes. Also, the development of an objective, systematic outcomes framework will form the basis for future LSM studies in the field. We aim to analyze the interactions between LSM outcomes, explore its diverse determinants, and identify senior travel phenotypes. We hope to develop interdisciplinary policy−driven interventions to ultimately improve the quality of life in older adults.

## Introduction

Life spaces (LS) are areas through which people move over a specified time period [1-3]. More recently, the study of LS incorporates active aging concepts [3-7]. Active aging models optimize opportunities for health, participation, and security to enhance quality of life (QoL) in older adults [8,9]. Accordingly, the descriptions of LS also include these opportunities and environmental experiences beyond simpler spatial quantifications. Thus, the movement within these activity spaces is termed life-space mobility (LSM). A full, contemporary definition of LSM is the movement of people over time and the total areas through which they move to achieve life goals of health, employment, security, and participation for active aging. This movement is often described as the individual’s mobility capital within these envelopes [10-13]. Examples of activities carried out in particular LS are dining, shopping, recreational, health care, social, or religious activities, and these are subsequently recorded into trip or travel diaries in LSM studies [11,14-16].

LSM is an important biomarker and determinant of population health [3,17-19]. Restricted LSM is associated with higher mortality risks and reduced QoL [5,10,20,21]. It also predicts the development of geriatric syndromes including dementia and falls [5,10,22,23]. Diminished LSM is further linked to future hospitalizations, readmissions, and increased risk of nursing home admissions [24,25].

**Figure 1.**
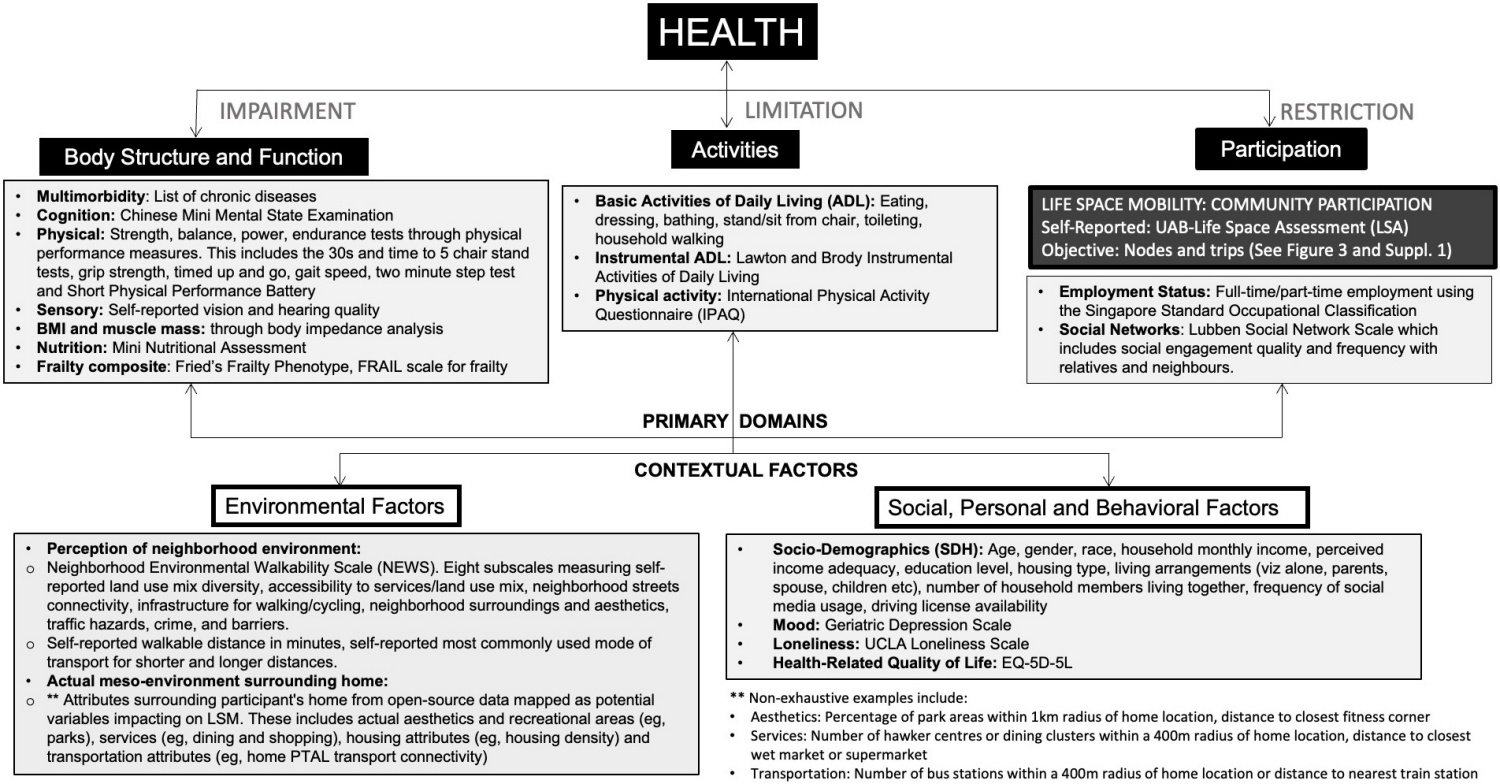
Life-space mobility (LSM) is a participation measure in the WHO International Classification of Functioning, Disability and Health (WHO-ICF) framework. Assessments performed in the Elderly Activity Life-Space Envelopes (EASE) map to the WHO-ICF domains and illustrate the breadth of the potential factors that may impact LSM outcomes. PTAL: public transport accessibility level; UAB: University of Alabama; UCLA: University of California, Los Angeles.

Multiple varied factors impact LSM itself. The WHO International Classification of Functioning, Disability and Health (WHO-ICF) provides a comprehensive framework, whereby LSM can be considered a measure of participation [26-29]. LSM represents not merely mobility capacity but actual community mobility performance [29-31]. Many diseases, impairments, activity limitations, and environmental, social, and personal factors can restrict LSM (Figure 1) [7,8,10]. Examples of influential diseases include stroke and hip fracture and key impairments such as declines in hearing, strength, or cognition. Poor performance on the activities of daily living can restrict LSM, as can limitations in participation, such as lack of employment. Both environmental contextual factors such as lower neighborhood walkability and personal contextual factors such as poor social network or loneliness can also reduce LSM.

The most established measure of LSM is the self-reported University of Alabama at Birmingham Life-Space Assessment (UAB-LSA) instrument [2,10,32]. Recently, emerging technologies in geographical information sciences (GIS) also provide more detailed and objective measures of LSM [3,9,13,33]. These include smartphone- or wearable-based advanced GPS platforms and accurate, open-source application programming interfaces (API) for environmental mapping [6,16,34].

For the reasons above, the study of LSM is a broad interdisciplinary science in health and medicine. It further integrates diverse sciences including sociology, geography, computer sciences, architecture, urban planning, and the environmental sciences [6,8,15].

There is a significant gap in the literature as contemporary LSM research is conducted mainly in scientific silos and published on diverse platforms. For example, most LS studies in medicine or sociology do not include geographical or built environmental attributes [8,21,27,35-37]. This reduces the impact of the transdisciplinary nature of LSM sciences on both risk assessments and eventual interventions.

The Elderly Activity Life-Space Envelopes (EASE) project is a large interdisciplinary mixed methods study. We first developed a set of LSM outcomes based on biopsychosocial and environmental constructs that underpin the study. The first phase included a large, comprehensive quantitative cross-sectional study on LS and their determinants in older adults. Selected participants from this cohort subsequently underwent an ethnographic qualitative study exploring their travel behaviors. Subsequently, phase 2 integrated phase 1 findings and comprised community design workshops and comparative testing in community locations of the built environment and space usage patterns to provide translational recommendations to improve LSM.

We focus on the main quantitative study methodology in this study. The overarching aims are to determine why, where, when, and how older adults travel in their LS and the health, social, and environmental determinants of these spaces.

In line with national health and urban priorities, we developed 3 primary hypotheses. First, age and LSM are inversely related [13,15,19]. Next, that a state of prefrailty or frailty results in diminished LS [10,38,39], and third, that the participants’ home (housing) typology results in significantly different LSM [40,41].

## Methods

### Ethical Considerations

The SingHealth Centralized Institutional Review Board reviewed and approved the ethics of this study (CIRB Reference Number: 2021/2566). Interviewees signed informed consent forms before participating in the study. All participants were given a 20-SGD (US $15) shopping voucher for finishing the initial in-person assessment and an additional 130 SGD (US $100) on completion of the 14-day e-travel diary [5,16].

### Sampling Frame and Sample Size

We recruited inclusively to capture the broad diversity of mobility among community-dwelling older adults in the Central and Eastern regions of Singapore. The key inclusion criteria were minimal. Participants had to be 50 years old or above and reside within the specified geographical area. This geographical boundary was necessary to concentrate and streamline the analysis of environmental attributes rather than having participants scattered countrywide. In addition, SingHealth is the largest health care provider in Singapore, and by working through its community partner arm, these regions fall within the SingHealth catchment area. This facilitated the necessary publicity and center liaison work while enabling us to reach out to a significant proportion and sufficiently wide distribution of Singapore’s older population described further below.

We did not specifically restrict participants based on their existing level of community mobility, aiming to capture the full spectrum of LSM ranging from older adults with high mobility to older adults with frailty. Exclusions were refusal or the inability to complete a consecutive 14-day period for GIS tracking and medically unstable participants, for example, older adults with poorly controlled medical, psychological, or behavioral issues.

We first performed a sample size calculation utilizing the UAB-LSA Instrument with age as the primary correlate. The correlation between increasing age and LS, based on a separate cohort of about 800 older adults recruited at community-based senior centers, was −0.21 [26,39]. Comparatively, in a similar study of 1000 older adults in the United States, this is approximately −0.36 [42]. Thus, power calculations indicate that with a sample size of 783, there will be 80% power (*β*=.20), with a type 1 error rate of 5% to detect a correlation as small as 0.1 between age and LS.

We also factored in a 10% drop-out rate to provide a buffer as participants may not complete the full 2-week period for a valid participation count. Thus, a total recruitment target of 900 older adults was reasonable and practical given our prior experience with community-based research [26,39,43].

For these 900 older adults, we performed quota sampling, that is, the selection of participants based on preset attributes into representative subgroups. These attributes were aligned with the primary hypotheses as having the largest impact on LSM and agreed by consensus within the interdisciplinary research team and supporting agencies.

These sampling categories are as follows: (1) two age brackets of 50 to 62 years old and 63 years or older, with a ratio of 40 (“younger-old”) to 60 (“older-old”) in the subgroups. The 63-year-old cutoff corresponds to the retirement age of Singapore citizens and is an age proxy for employment status [44]. (2) Public versus public housing with participants in a 20 (private):80 (public) ratio. This is in line with the nationwide housing type distribution [40]. (3) Frailty status categorized dichotomously by prefrail and frail versus robust seniors. We aimed for a distribution ratio of 30 prefrail and frail: 70 (robust) participants, in line with current Singapore community prevalence rates estimates [26,39,45].

To achieve this broad yet population-representative cohort, we deployed a comprehensive, 2-pronged strategy to ensure wide reach across the SingHealth catchment area. The first prong involved direct recruitment, primarily executed through online registration forms, to capture independent and digitally connected older adults. The second prong focused on community outreach and leveraged collaborative partnerships established through the multi-institutional research team and SingHealth’s community partner arm. This strategy involved working with more than 10 dedicated community partners, such as senior activity centers, which provided essential access to older adults who may not be as digitally active. Wide publicity was further achieved via the investigators’ institutional and community networks, using custom-designed publicity media (Multimedia Appendix 1). This combined approach successfully supported the quota sampling method to ensure representation aligned with the primary hypotheses and local community prevalence rates.

All successfully recruited older adults participated in a community-based, in-person comprehensive assessment, followed by an electronic travel diary tracking of LS over 14 days. The 14-day recording of the e-travel diaries was decided by several factors. Previous research recommends at least a 7-day consecutive period to capture 2 weekend days [7,11,46]. Some investigators suggest that a 14-day period is ideal to account for within-person variability, especially for the nodal extent measures [9,16,47]. A 2-week period may also better corroborate to the UAB-LSA self-reported measure [33]. This balances against the significant participant burden, compliance, and privacy concerns in longer time frames [7,18,30,34].

### In-Person Assessment Phase

#### Overview

**Figure 2.**
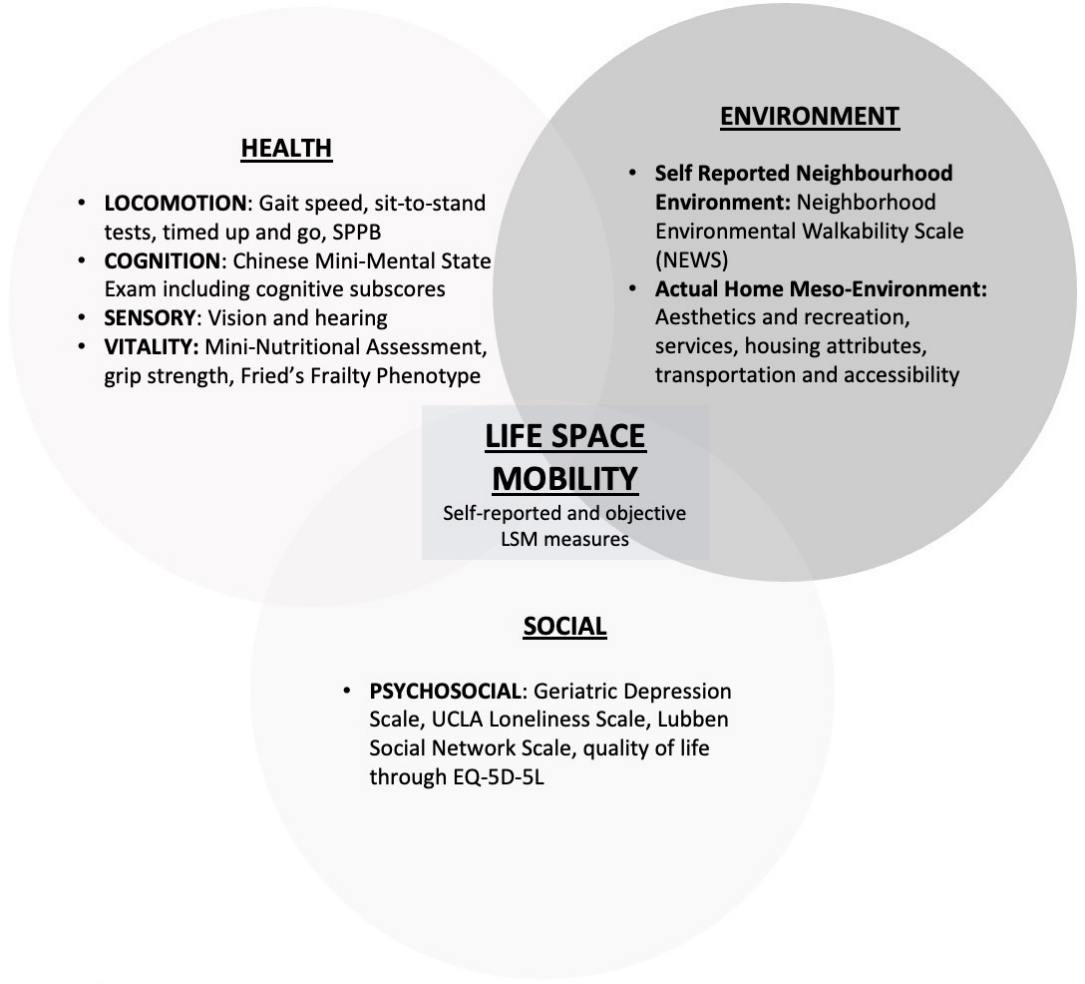
Life-space mobility (LSM) as the interface between the WHO-Intrinsic Capacity construct and the external environment. Intrinsic capacity encompasses the 5 key domains: locomotion, cognition, psychosocial, sensory, and vitality. Assessments performed in Elderly Activity Life-Space Envelopes (EASE) map into intrinsic capacity domains. SPPB: Short Physical Performance Battery; UCLA: University of California, Los Angeles.

This phase comprised a comprehensive multidomain geriatric assessment (MDGA) including a set of physical performance measures [39]. This custom-designed assessment itself was developed based on the overarching biopsychosocial constructs of the WHO-ICF, Frailty, and Intrinsic Capacity (Figures 1 and 2) [26]. These variables were also selected based on their prior usage in previous LSM literature and interventional potential to increase LSM [8].

This also drew on our previous work in Individual Physical Proficiency Test for Seniors, a community-based frailty and sarcopenia screening platform that has been implemented and tested for feasibility [26,39,43,45]. We modified the Individual Physical Proficiency Test for Seniors MDGA for EASE to also include environmental determinants, while keeping the length of assessment itself manageable for both the researchers and the older adults. Figure 2 shows the domains and variables in the MDGA underpinned by the intrinsic capacity–external environment construct.

#### University of Alabama at Birmingham’s Life Space Assessment

The UAB-LSA was used to measure self-reported LS in this study [32]. It is the most widely used questionnaire worldwide and is internationally validated [2,10]. It provides a composite measure by combining the level of life space attained, degree of independence, and frequency of attainment over the past 4 weeks. The levels have been adapted for relevance to the local context as more than 95% of the population lives in public or private apartment blocks [26,39]. These are as follows: (1) level 1: rooms of the home, other than the bedroom; (2) level 2: an area outside the home, but within the block; (3) level 3: places in the neighborhood, other than the block; (4) level 4: places outside the neighborhood, but within their residential estate or township; and (5) level 5: places outside their residential district or town.

The maximum score is 120, and scores of less than 60 are categorized as restricted LS with a difference in 10 points being significant [35,48]. Recent summative reviews indicate that the global LSA is 66.8 with 42% having restricted LSM [24].

#### Frailty State

We aligned EASE with the National Frailty Strategy for Singapore to provide translational relevance [38,49]. Accordingly, we screened for frailty with the Fried’s Frailty Phenotype and the FRAIL (Frailty, Resistance, Ambulation, Illness, Loss of weight) scale. The 5 components of the Fried’s Frailty Phenotype are weight loss, exhaustion, physical activity, walk time, and grip strength [50]. The FRAIL scale consists of the 5 questions on fatigue, resistance (strength), ambulation, illness (multimorbidity), and loss of weight [51].

For both of these instruments, the summative score of 0 indicated a robust status, 1‐2 points indicated a prefrail status, and 3‐5 points indicated a frail status. Where available, the criteria were adjusted based on established local or Asian norms [43,45].

#### Quality of Life

Health-related QOL was measured using the EuroQol 5-Dimension 5-Level [52]. Five dimensions are assessed, that is, mobility, self-care, usual activities, pain or discomfort, and anxiety or depression, with 5 response levels per dimension (1=no problems to 5=unable to or extreme problems).

Five-digit profiles, ranging from 11111 (full health) to 55555 (worst health), were generated by concatenating scores across the dimensions. These 5-level profiles were then converted to index values using a crosswalk calculator, applying Singapore’s 3-level value set [53,54]. Participants also rated their overall health on the EuroQol Visual Analogue Scale, a 0‐100 visual analog scale (worst to best imaginable health) [52].

#### Chinese Mini-Mental State Examination

The Chinese Mini-Mental State Examination is an established screen of general cognitive function that is locally validated and deployed in LSM studies [49,55,56]. It consists of 28 questions assessing the 6 cognitive domains of orientation, registration, attention, calculation, recall, language, and praxis. One point is given for a correct answer, and a Chinese Mini-Mental State Examination score of <21 indicates cognitive impairment.

#### Geriatric Depression Scale-Short Form

The Geriatric Depression Scale-Short Form is a validated, sensitive, and specific 15-item self-report questionnaire to screen for depression in older adults [14,57,58]. It evaluates the emotional and cognitive symptoms rather than the physical signs of depression. “Yes” responses to negatively worded questions and “no” responses to positively worded questions are given a score of 1. A total score of 5‐9 points suggests depression, and ≥10 was almost always indicative of depression.

#### Lubben Social Network Scale

We used the Lubben Social Network Scale-Revised instrument to measure the strength of social network and assess social isolation [59,60]. This 12-item scale measures the network size, closeness, and frequency of contact with family members and friends. The score for each item ranges from 0 to 5 with a maximum score of 60. A score of <20 indicates restricted social networks and a high risk of social isolation.

#### University of California, Los Angeles Loneliness Scale

Loneliness was assessed using the University of California, Los Angeles 3-item loneliness scale [20,58,60,61]. It measures 3 dimensions of loneliness: lack of companionship, being left out, and feeling isolated. A 5-point frequency scale, ranging from “never” (0 point) to “always” (4 points), was used to evaluate responses to the 3 items. Higher scores indicated a higher level of perceived loneliness.

#### The Neighborhood Environment Walkability Scale

The Neighborhood Environment Walkability Scale (NEWS) is the most used measure of the perceived neighborhood environment worldwide [62,63]. We adopted the NEWS-Abbreviated (NEWS-A) version for practicality as it has been used successfully in older populations [64]. The NEWS-A assesses the key environmental dimensions of a person’s subjective experiences of their neighborhood environment.

These domains are accessibility to services, land use mix (the diversity of destinations), street connectivity, infrastructure for walking, neighborhood esthetics, traffic, and personal safety [15,64]. All items in these domains are rated on a 4-point Likert scale ranging from 1 (“strongly disagree”) to 4 (“strongly agree”). Due to the length and complexity of the questionnaire, responses of “do not know” were allowed.

#### International Physical Activity Questionnaire-Short Form

Weekly physical activity was estimated using the International Physical Activity Questionnaire-Short Form, which weights the time spent in each activity intensity with the estimated metabolic equivalent of task (MET) [49,65,66]. MET minutes per week were calculated by multiplying the respective MET values (“vigorous”=8.0, “moderate”=4.0, and “walking”=3.3) with the number of minutes the activity was carried out and the number of days the activity was carried out. Participants were asked to estimate the number of hours they spent sitting on an average weekday serving as an indicator of time spent in sedentary activity [65]. The products across the 3 levels of activity were summed to derive the total MET minutes per week.

#### Physical Assessments and Performance Measures

Physical assessments performed include the BMI and muscle mass through multifrequency segmental bioelectrical impedance analysis (MC-780 MA P, Tanita). Physical performance measures included the handgrip strength test, 5 times and 30-second sit-to-stand tests, Timed Up and Go test, 10-m walk test for gait speed, Two-Minute Step Test, and the Short Physical Performance Battery [8,21,34,37,67]. Cut-off thresholds for these measures prioritized local and Asian norms, frailty, and sarcopenia guidelines where appropriate and available [26,39,43,49,68].

### Objective Life Space Measures

#### Overview

Objective LSM measures through GIS systems have distinct advantages over self-reported LSM. These include enabling assessment of older adults with cognitive challenges, reducing recall bias, and documenting the duration and type of travel modes and the places participants go to [1,18,34,69].

However, there is no consensus on what constitutes these objective measures [47]. Classification frameworks exist; however, these constructs tend to be technical with terms such as nonexclusivity and stability [1,3,5,9,33,70]. This can be challenging to investigators in fields such as health and sociology.

**Figure 3.**
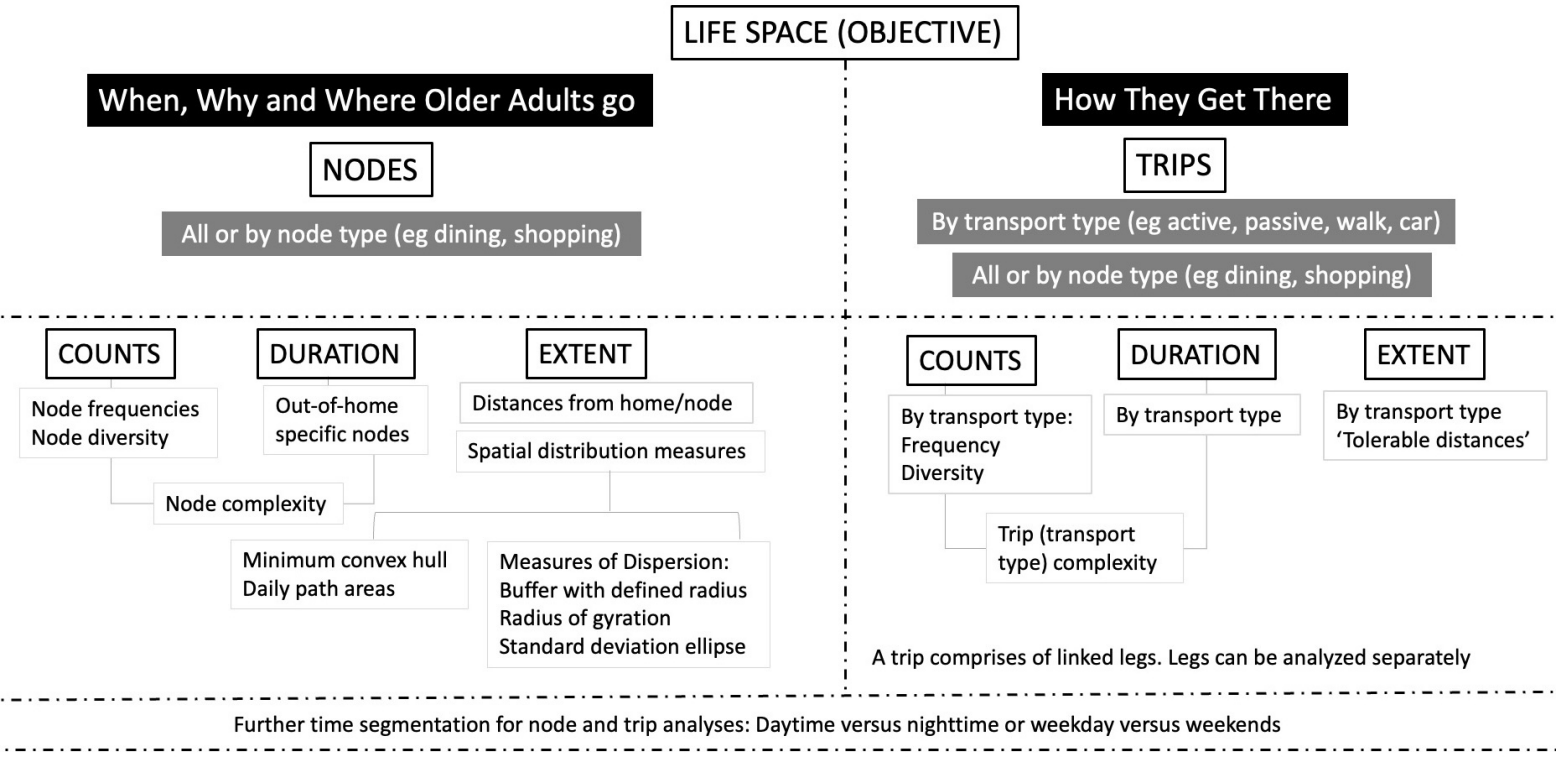
Framework of objective geographical information sciences (GIS) life-space mobility (LSM) measures based on Elderly Activity Life-Space Envelopes (EASE) primary aims of “where older adults go to” and “how they get there.”

We developed a focused, easy-to-understand framework of life-space GIS indicators to bridge the interdisciplinary nature of the EASE project (Figure 3). We considered measures with availability of comparative data in the literature, their known impact on health or relation with sociodemographics, their importance in future interventions, and the capacity of present-day smartphone-based apps to obtain these data [1,18,30,71,72]. Figure 3 is a brief description of this framework.

We first classified measures into the primary aim of this project articulated previously of “where do older adults go to” and “how do they get there.”

**Figure 4.**
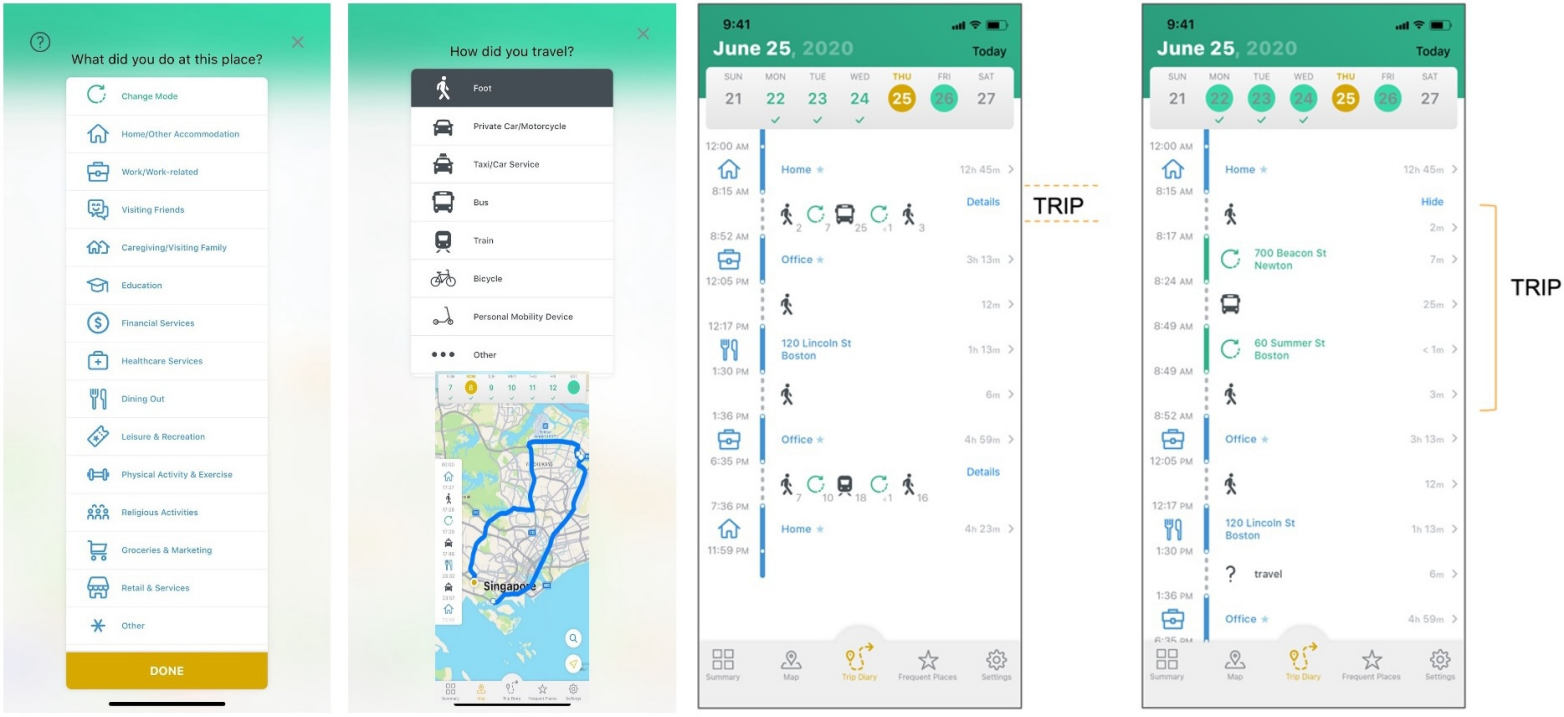
Trip diary interface. Snapshots of the customized X-ING Future Mobility Survey (FMS) app showing selection for activity or nodes, selection for travel type, trip, and trip legs.

For measures in the first group, we adopted the neutral term “nodes” to describe where the participants went or what they did there. We use the term “nodes” consistently throughout the EASE study for this purpose. Nodes have been variously termed attributes, opportunities, staypoints, stops, locations, and activity points in other research based on their underlying study aims or construct [6,16,33]. We defined 14 types of nodes for this study based on large local surveys, existing literature, and conceptual models categorizing nodes by activity importance [5,7,11,14,33,40,53]. This list of nodes is given in Figure 4.

#### Node Characteristics

Node measures are divided into counts, durations, and extent. They can be analyzed as a whole or by a specific node type. Node counts include the *frequency* of the nodes visited and the count of the number of different node types (node *diversity*) visited [1,18,33,53]. The node *duration* is the time spent at a node [16,31,53]. A composite of these measures is node *complexity*, partially based on entropy concepts [33,34,73]. Higher complexity indicates greater variability in either frequency, type, or duration spent at the nodes when time periods are compared.

The node extent refers to the distribution of these nodes in the geographical space. This is measured by distances from home or between the nodes. Subsequently, a count of nodes can be done within a defined radius from home or a specified (eg, work) node [5,74,75]. Weighted or unweighted spatial metrics that encompass the nodes can also be obtained, including the minimal convex or concave hull, standard deviation ellipse, daily path area, and radius of gyration [1,7,13,19,30,56]. Additional detailed descriptions of objective LSM measures are available in Multimedia Appendix 2.

#### Trip Characteristics

In the second group of measures, we defined the term “trip” to represent how older adults get to the nodes. Trips are variously termed moves, excursions, trajectories, or paths in the GIS literature, but we believed “trips” are clearer and more distinctive in interdisciplinary research [1,9,33,34].

Trips are analyzed by their type (mode) of transport. A single trip can be further divided into trip legs with different modes of transport. A common example is a 3-leg trip to a shopping center comprising a walk from home to a bus stop, then a bus ride, and finally a walk from the bus stop to the shopping center. Like nodes, these trips can be analyzed as a whole or to specified node types. Individual legs within trips can also be analyzed.

Trip measures are also divided into counts, duration, and extent. Trip count would be the frequency of trips on a specified type of transport. Trip count (diversity) is the count of different transport modes used [1,72]. The trip duration is the length of time spent on a trip by a transport type [6,19]. We distinguished between active and passive transport modes as there are better health outcomes when more time is spent on active modes like walking and bicycling [5,6,33,76].

A higher trip complexity then represents a greater variability in trip frequency, type, or duration by transport modes between time periods. The trip extent refers to the trip mode as a function of the node extent as described above [5,9,16,77]. For example, walking is the most common mode of transport within 2 km from home. In other words, 2 km is a tolerable walking distance [6,31].

For all the trip and node measures, further insights can be obtained by segmenting data between day or night, or weekday or weekends [19,20,33,69,71]. Both linear and actual (GPS) distances were collected in EASE. Although GPS distances are more accurate, this is often limited by technical challenges due to reception issues in built-up or enclosed areas, or hardware issues like battery life on smartphones [75].

### Smartphone Travel Diary Survey System

Our GIS application, X-ING, is based on Mobile Market Monitor’s Future Mobility Survey (FMS) architecture [16,78,79]. This is a smartphone-based, automated mobility data collection system that has been field-tested and deployed worldwide to measure LSM and community travel patterns [16,77,79]. It is compatible with Android and iOS systems and has been designed to collect accurate travel data from the phone sensors in a nonintrusive manner while minimizing battery consumption [78]. Briefly, the FMS system consists of 4 interconnected components:

1. The X-ING app that unobtrusively records and integrates the relevant GIS sensor data. This includes GPS, GSM cell tower, Wi-Fi network, accelerometer, and barometric data acquired in the user’s smartphone.
2. The dedicated server hosting the database as well as the data processing and machine learning algorithms. To maintain data robustness where GPS signals may be weak (eg, indoors or underground), these algorithms perform multisensor fusion, integrating all raw GIS sensor data (GPS, GSM cell tower, Wi-Fi, and accelerometer) for continuous and accurate location inference. These algorithms detect stops (nodes) and infer travel modes based on the user’s GIS sensor data, local contextual data such as public transport network locations, and the user’s previous validated trip diary.
3. Trip diary interface within the X-ING app that allows users to verify their daily travel including mode of travel, node activities, and locations (Figure 4). The interface also prompts users to provide supplementary trip details such as whether the participant was the driver or passenger in car transport modes.
4. The data management system used by the researchers to monitor, manage, and quality control incoming data throughout the entire study duration.

The X-ING app was customized for the EASE project, taking into consideration local geography, transportation networks, and GIS infrastructure [16]. All data were encrypted and secured both in transmission and within storage and anonymized in compliance with Singapore’s Personal Data Protection Act and the institutional review board [80].

The details of the data processing are provided elsewhere, and a summary is as follows [16,77-79]. Raw data were first filtered to remove clearly invalid points or nodes that occur, especially when there is a paucity of GIS sensor data. Examples include distance errors or high altitudes that are incongruent with Singapore’s geographical attributes.

Next, candidate stops are generated through a clustering algorithm where GPS data indicate that participants have been within an area of a 50-m diameter for at least 1 minute. These stops are then verified against sensor place signatures that the user visits frequently. New candidate stops are checked against these signatures, and if a match is found, the stop is moved to the recorded home, office, or nodal activity location. Stops are further consolidated and merged by integrating other sensor information including GSM cell tower, Wi-Fi, and accelerometer data to eliminate phantom trips or location jumps caused by data noise. For example, Wi-Fi signatures are used to determine if participants are in the same indoor location when GPS data are unavailable.

Movement trajectories were then segmented into active or passive transport modes by integrating smartphone data sources like accelerometry and GPS to provide better precision to the transport type. The app further incorporates information of local transport network data (eg, to observe that routes coincided with train routes or bus stops) and user-validated input. Algorithms include decision trees to classify shorter stops within a trip into potentially important stops like changing of travel modes or pick-up or drop-off activities versus unimportant ones.

These processed data were then presented on the trip diary interface (Figure 4) where the participant confirms the node locations and trip-related information including the type of vehicular transport used. The FMS platform has learning attributes; thus, the participant burden to verify the data diminishes as the algorithm learns LSM patterns based on its existing databases and preceding user validations. Where possible, we also addressed ambiguity by recoding activities and travel modes. Specifically, we examined all linked free-text entries when the “Others” option was selected, created a free-text dictionary, and successfully recoded 2086 (34.2%) of the 6084 free-text entries across the dataset to the appropriate travel mode or activity. Backend data monitoring was performed, and check-ins were done if there were no valid data for 3 or more consecutive days [33].

For older adults who did not have, or declined using, smartphones, manual paper logs were used. Researchers then input these data into a web-based diary entry system, which is subsequently integrated into the same FMS project database to ensure data consistency.

### Home Locations and Built Meso-Environments

#### Overview

The home location was defined as the majority of overnight stays at a node [9]. In EASE, home nodes are represented by the local 6-digit postal codes. Validation checks were done by first geolocating participants’ self-declared postal code home address (self-declared node, SDN) with the OneMap API [81]. We next determined the GPS location (GPS node, GPN) of the participants at 0300 hrs (defined as overnight stay point) on the majority of nights [9]. We subsequently evaluated the similarity of each participant’s SDN and GPN location by using a set of guidelines of average block dimensions and spacings to account for deviations [82]. All GPN or SDN deviations more than 100 m were visually inspected on OneMap, and GPNs were snapped to the closest postal code location if this was deemed to be the likelier home location. The large majority (98.7%) of the final home points were SDNs [34].

We next proceeded to describe their unique meso-environments around the participants’ home up to a 1-km radius buffer by loading open-source environmental attribute layers into QGIS [6,70,76]. We prioritized using sources based on authoritativeness and accuracy [75].

First, we included GIS data from the government agencies. These include OneMap, Data.gov.sg, and the LTA DataMall [81,83,84]. Next, we applied layers from established GIS providers, and these were OpenStreetMap (through Quick OSM) and the Google Maps API. Finally, any further data needed were obtained from known reputable local websites, particularly data on private housing attributes.

We used OneMap as the basemap and applied 4 groups of environmental attributes as vector layers, merging where appropriate.

#### Esthetics, Recreation, and Activity Areas

This included National Parks and Reserves (GovTech, Singapore 2025), complemented by “Parks” and “Playgrounds” [85]. We further added layers of activity areas and fitness corners in the community [83,85].

#### Services

Dining vector layers include hawker and food Centers [83] and dining establishments [85]. Marketing layers include (wet) markets and supermarkets [83,85]. Shopping mall vectors were also layered, as were senior activity centers [14,76,85].

#### Housing

We obtained public and private apartment buildings and landed housing attributes [83,85]. Consistent factors documented were the age of these buildings and the number of dwelling units within a building reflecting housing density [76].

#### Transportation

These metrics include geospatial vectors of public transportation location such as bus stops and mass rapid transit train stations [84]. We also added a road layer to calculate both connectivity and density metrics. This layer contains both vehicular road networks, as well as designated cycling paths and park connectors vectors [83,84].

#### Accessibility

We determined the Public Transport Accessibility Level (PTAL) for each participant’s home node. The PTAL itself is a well-known metric of transport connectivity, and we used an established algorithm adapted for the Singapore context [86]. The determinants in the PTAL score include the walking distance from home to bus stops and mass rapid transit stations and the count and frequency of different buses or train services at each of these transport nodes [86].

### Vertical Life Spaces

We also incorporated advancements in smartphone technology to record vertical LSM [87]. We utilized an algorithm that integrated both GPS and barometer sensors, whereby the GPS signals identify horizontal location entry points, while the barometric data detect relative changes in atmospheric pressure during rapid vertical movements. This building infrastructure–independent method has shown good accuracy rates previously [87]. We primarily focused on vertical trips at home locations in this pilot exercise to balance the significant burden required to validate many vertical movements in other locations such as workplaces or shopping malls.

### Standardization and Quality Assurance of the 14-Day Life Space Tracking Window

To ensure the consistency, integrity, and quality of the objective LSM data collected, the 14-day tracking period was strictly standardized across the 3 phases of preparation, in-field data collection, and final data standardization.

In the preparation and commencement phase, the tracking period officially commenced the day immediately after the in-person comprehensive baseline assessments. This ensured a consistent starting point and minimized recall bias. All patients underwent a mandatory training session by the research team members on the use of the X-ING smartphone app or the corresponding manual data log, including assistance with installation and a show-back demonstration on its use.

During the in-field data collection phase, there were 2 key quality assurance measures. First, there was the integration of daily prompted recall via the FMS/X-ING system. At the end of every day, all participants were prompted and required to validate the automatically inferred data in their trip diary, including node locations and travel modes as described previously. This validation process served as the essential check to capture ground truth information that the X-ING app inferred. Second, the research team rigorously contacted participants every 2‐3 days if compliance appeared low or if data quality issues were flagged by the backend system. For older adults who could not or had difficulty using the app, a paper-based travel diary was provided to ensure continuity of data collection and actively prevent missing days.

The final standardization phase involved rigorous backend processing to ensure 14 full days of valid travel logs for inclusion in the cohort for analysis. This included invalid day removal where days were flagged as invalid if participants did not carry their smartphones during community travel, made trips outside of Singapore, or voluntarily declared the day as invalid. Further, only participants who completed the full 14 days of valid electronic or paper-based travel diary data were retained for the final analysis of objective LSM measures. Those with insufficient data were ultimately withdrawn from the study cohort used for these specific analyses.

### Statistical Analysis

A combination of coding and statistical tools was used in EASE. R version 4.3.2 (R Core Team) and SPSS version 29 (IBM Corporation) were the primary quantitative analysis software, and QGIS version 3.34 was the primary spatial software used [13,30,56,71]. Python version 3.12 (Python Software Foundation) and its PyQGIS variant (QGIS Development Team) were used to extract, aggregate, and transform data from multiple sources, develop spatial metrics, and analyze spatial data [13]. As this paper focuses on the background and methods in EASE, we primarily employ descriptive statistics only. This includes means (SD) and medians (IQR) to provide overviews of central tendencies and data variability.

For the 3 primary hypotheses on relationships between age, frailty status, and housing typology on LS, we will first define the LS outcomes, which include both self-reported (UAB-LSA) and objective GIS LSM measures (Figure 3). We will initially explore these associations using bivariate analysis, such as Pearson correlations (for continuous variables like age) and independent samples *t* tests or analysis of variance for categorical variables (frailty status and housing typology) against the LS outcomes.

To test the hypotheses while controlling for confounders, we will develop multivariate regression models. Multiple linear regression will be the primary method for continuous LSM outcomes (eg, LS spatial measures such as mean convex hull or standard deviation ellipse), while for count data (eg, node frequencies, trip counts), a generalized linear model, such as Poisson or negative binomial regression, will be employed to handle the non-normal distribution of these variables.

Covariate selection will be guided by both extant literature and clinical or multidisciplinary consensus, drawing from the comprehensive set of EASE variables categorized by intrinsic capacity (eg, physical performance measures like grip strength) and external environment (eg, neighborhood walkability scores from NEWS-A), or by health, social, and environmental characteristics. Specifically, models will be adjusted for essential confounders including important sociodemographics (eg, education, income, private car availability).

Prior to model interpretation, assumptions will be rigorously assessed, including analyses of the normality of the residuals and linearity of the relationship between continuous variables. Variables will be mathematically transformed if distributional or linearity assumptions are substantially violated. Given the 3 primary, prespecified hypotheses, a 2-sided significance level will be set at *P*=.05.

Further statistical methods will depend on the specific hypotheses of future studies.

### Quality of Trips and Life-Space Activity

We also acknowledge the importance of understanding the subjective experience and quality of these trips—often referred to as semantic attributes of mobility or captured via Ecological Momentary Assessment in travel diaries [7,15,16]. However, for the projected large quantitative sample of over 1000 participants, requiring detailed, self-reported text descriptors on smartphones for trip purposes, meaning, or satisfaction for every journey poses an onerous burden on older adults, potentially increasing recall bias, non-response rates, and necessitating substantial staff training and daily support. Instead, our quantitative protocol prioritized accuracy and objective data capture of transportation and nodal activity by requiring participants to validate automatically inferred trip information daily, minimizing self-reported text input as described above.

The issues of trip purpose and quality, and the meaning of places (placemaking) were comprehensively addressed by the study’s mixed methods design through a significant qualitative component that used ethnographic methods. A subset of 60 participants from the main cohort was purposively selected based on the key quantitative stratifications (age group, housing type, and health or frailty status) to ensure representation across the main study hypotheses. These participants underwent a 2-session qualitative assessment: a semistructured in-depth interview involving cocreation of a mental map to explore the meaning of places and travel inclinations, followed by a “go-along” interview. During the “go-along” interview, researchers accompanied the participants on a self-selected typical route, using a combination of participant observation and dialogue to document the real-time social context, pleasurable attributes, and environmental facilitators or barriers that contribute to the quality and satisfaction of their community mobility. This in-depth approach, analyzed using principles of thematic analysis, provides a rich, granular, and controllable dataset on the subjective experience of travel that the high-volume quantitative travel diary could not achieve. It also strongly complements the quantitative LSM measures by offering contextual, explanatory data, for example, identifying environmental barriers for older adults or older adults with frailty with restricted LSM [5,31].

## Results

We recruited 1131 older adults at 7 community-based sites in Singapore. A total of 13 (1.1%) participants did not complete the 14-day electronic or paper-based travel diary and were withdrawn, leaving 1118 successfully completed participants who were included in this study.

The large majority (n=1062, 95%) successfully documented their travel diary on their smartphones, with the rest using paper-based travel logs. A total of 88,166 node points were recorded, excluding nodes due to a change in transport mode between trip legs, but including home nodes. There were 76,741 trips and 106,323 trip legs documented through the e-travel diary platform. Valid vertical LSM data were obtained from 228 participants.

The average age was 63.8 (SD 7.6) years, and there were 634 (57%) “Old-Old” older adults. About two-thirds (n=760, 68%) were female, 74% (n=1025) were of Chinese ethnicity, and 68% (n=759) were married. A significant proportion of the cohort was still either in part-time (n=221, 20%) or full-time (n=257, 23%) employment. The most common housing typology was public apartments (n=842, 75%). Around 62% of the older adults had a valid driving license. Healthwise, 326 (29%) were prefrail or frail by the criteria listed above. Demographic distribution data are available in Multimedia Appendix 3.

On average, the linear distance from home to the nearest dining option was 118 (SD 92) m, 1.8 (SD 1.5) km to a hawker center, 309 (SD 220) m to a supermarket, 688 (SD 422) m to a shopping mall, and 713 (SD 603) m to a senior activity center. The median PTAL score was 4 (IQR 3). The mean score of the UAB-LSA LSM was 90.2 (SD 18.13), and average convex hull was 101.7 (SD 84.6) km^2^.

## Discussion

To the best of our knowledge, EASE is the most comprehensive, large-scale LS study that we are aware of with its holistic assessment of multiple domains and the range and complexity of LSM measures. We had included both objective and perceived measures of LSM and believe they complement each other in providing different information on activity spaces. The lack of significant associations between GPS and self-reported data suggests that they measure different LSM dimensions [18,34,53,66].

The EASE study’s dataset is also wide-ranging and rigorous due to its foundational underpinning of potential predictors of LSM through contemporary aging and disability biopsychosocial frameworks. This notably includes the WHO-ICF; abbreviated comprehensive geriatric assessments models (MDGA); and population health constructs encompassing social, behavioral, and environmental determinants [6,8,15,28]. This meticulous approach ensures alignment with critical population health issues such as frailty, sarcopenia, disability, physical fitness, and intrinsic capacity in older adults [26,71]. Consequently, our dataset positions itself as a valuable minimum dataset, aligned with national priorities, to advance understanding and interventions in these domains.

In this light, we had considered the time required to complete the in-person assessment prior to participant fatigue. Several LSM GPS studies have minimal, if at all any, in-person contact [13,18,19,88]. However, the in-person assessment permitted the assessment of cognition, physical performance measures, and muscle mass for the health-related constructs [37,67,69]. Overall, participants took about 2 hours to complete this phase. We alternated the physical performance measures, questionnaire portions, and information about the app usage where necessary to retain focus and minimize fatigue. We believe that this assessment set was feasible and practical as all recruited participants completed the in-person phase.

The proportion of the older adults (n=13, 1%) who dropped out from the 14-day travel diary was significantly lower than the expected 10% buffer [18,34]. This positive finding reflects the effectiveness of several strategies and the cohort’s characteristics. The participants' general “tech-savviness” facilitated their engagement with the travel diary system, while the comprehensive in-person app demonstration reduced uncertainty and user errors. Open bidirectional communication was also pivotal [33]. Participants were encouraged to clarify concerns throughout the study, complemented by the research team’s proactive data management, including backend reviews and timely reminders to participants. The study’s methodological design, which incorporated tiered completion incentives, also contributed to the high retention rate [16,18,19,67]. Furthermore, the low dropout and missing data rate was a direct result of the study’s robust, multilayered quality assurance framework. This integrated system, combining the X-ING app’s automated algorithmic inference and daily participant-driven validation with rigorous human monitoring and data cleaning, effectively maintained data quality and completeness for the entire cohort, negating the need for complex, post hoc imputation methods for activity or travel modes.

Potential research areas include relationships between objective LSM measures (eg, trip length and dining nodes) or between self-reported and objective LSM measures (eg, between the UAB-LSA and the minimum convex hull) [18,34,53,73]. A second research avenue will investigate the demographic, health, social, behavioral, and environmental factors influencing LSM measures [47,53]. This would involve studying how various population health themes like aging, frailty, intrinsic capacity, mental health, and QoL relate to each other and impact LSM [1,20,31,53,71].

We also aim to develop participant LSM phenotypes and profiles through cluster analyses [33,73,79]. These include identifying characteristics of the older adults who predominantly travel during the day versus night, those who use various transportation modes, or those exhibiting specific travel node patterns such as home-work-home or home-dining-shopping-home [16,77,79].

Further, utilizing unique meso-environmental attributes derived from open-source data offers an unexplored but promising research opportunity. By adopting a “bottom-up” and “top-down” approach, researchers can investigate how individual movements within the environment (“bottom-up”) align with broader urban planning and transport policies (“top-down”) [76,89]. This includes exploring correlations between elements like the PTAL transport connectivity and actual LSM or assessing if housing density and road infrastructure relate to mental health aspects such as depression [6,7,75,86].

We hope to translate the work through important integrative themes. We highlight 2 important themes of walkability and food behaviors [13,47,76,90]. Walkability is linked to enhanced health and social outcomes, aligning with Singapore’s urban design goals of a walkable city and the WHO Healthy Cities blueprint [91]. Similarly, dining activities hold significant health and social implications, influencing government planning that promotes social dining spaces such as hawker centers [16,90]. The baseline dataset serves as a foundation for qualitative research and phase 2 exploration, ultimately guiding city planning interventions toward active aging and community well-being.

Finally, an advancement in EASE is the pioneering mapping of 3D LS as a further LSM outcome measure. In our study, only 20% (n=228) of the older adults had smartphones with barometers capable of measuring vertical movement, but this will increase with advancements of cell phone technology incorporating sophisticated sensors and algorithms [87]. This is particularly relevant in densely urbanized settings where LSM involves vertical movement within multifunctional buildings, a situation further intensified by the COVID-19 pandemic, which has blurred lines between home and workplace environments [91].

A potential limitation of the EASE study is its focus on older and community-dwelling participants, which may limit the generalizability of the findings to other demographic groups. However, this represents the tight balance against the (already) significant resources required to conduct such an extensive study. To mitigate this, the quota sampling frame allowed us to concentrate on the most relevant variables and ensure representation of the key characteristics within the cohort. Additionally, our focus on community-dwelling participants was intentional, as we aim to establish LSM measures as an effective screening tool before individuals develop adverse health outcomes. We could also have further included accelerometry data to measure step counts or physical activity [19,34,47]. However, we prioritized GIS information due to battery life limitations and the reluctance of participants to use another wearable [73].

Another established limitation is the reduced measurement accuracy of GPS systems for highly granular movement within dense urban infrastructure, basements, or underground transport systems, such as train tunnels. However, the methodology of the EASE study was deliberately balanced with its central research goal: characterizing LSM, which focuses on the extent and complexity of travel and activity between distinct community nodes (eg, home, parks, shops, clinics). Consequently, we considered the short duration of activity loss experienced in these sheltered areas a minor trade-off, which is offset by the robustness gained in accurately mapping the overall extent and frequency of out-of-home activity, which forms the core of LSM assessment. Furthermore, to address a common gap in 2D mobility studies, our comprehensive data fusion protocol incorporated signals from GPS, cellular networks, and Wi-Fi, in addition to barometric sensors, in capable smartphones to explicitly capture 3D life-space mobility, such as ascending floors within a building, and to supplement location data in areas where GPS signals are attenuated, like indoors or underground structures.

A further potential limitation of the EASE study is that the project is conducted entirely in Singapore, within its specific societal and environmental context. However, the core strength of this protocol is its high replicability and generalizability across diverse international contexts. This is achieved through the use of universal biopsychosocial constructs (like WHO-ICF, Frailty, and Intrinsic Capacity), deployment in a high-density urban testbed, the development of a novel and universally translatable objective construct for trip or node measures, and a commitment to sharing a comprehensive research toolbox with the global community.

The integration of big data into LSM studies like EASE presents both promising opportunities and significant challenges for future research [25,92,93]. Big data, characterized by its high volume, velocity, and veracity, enable a detailed analysis of LSM through GPS, accelerometry, wearable technology, RFID, and infrastructural sensors [16,19,20,34,58,88]. This can be further enhanced by integration with sophisticated social media platforms within the Internet of Things ecosystem [94]. The fusion of electronic health and social records with LS analytics through artificial intelligence also allows for precision medicine, providing personalized health interventions and comprehensive community participation profiling through self-biofeedback and risk monitoring [17].

However, the EASE research team also believed that we were approaching a ceiling on the amount and granularity of data participants would be prepared to give [66,88,95]. As LSM research becomes more pervasive, safeguarding privacy through coherent governance and legislation is paramount. Researchers have to navigate varying personal thresholds for data sharing, often leading to participation rejections and smaller sample sizes [71]. This is an evolving challenge that must involve the consensus between the interdisciplinary stakeholders in future LSM research.

In conclusion, the EASE study incorporates a comprehensive methodology, integrating LSM within population health themes through a holistic health, social, and environmental framework. By utilizing both self-reported and objective LSM measures, EASE not only deepens the understanding of LSM but also introduces an interdisciplinary language for objective LSM markers, enhancing cross-disciplinary communication and research integration. The feasibility and practicality of the study are highlighted by its low drop-out rate and use of open-source software and public data, providing a minimum dataset that establishes a robust foundation for future research. This framework opens several avenues for innovative exploration, from analyzing diverse determinants that impact LSM to developing practical, policy-driven interventions. We hope that the methodology and future findings in EASE significantly contribute to the development of LSM sciences, and further to active aging, urban planning, and public health, ultimately aiming to improve the QoL of older adults.

## Supplementary material

10.2196/79308 Multimedia Appendix 1 Publicity materials for Elderly Activity Life-Space Envelopes in the 3 national languages of Singapore.

10.2196/79308 Multimedia Appendix 2 Table of objective geographical information sciences life-space mobility (LSM) measures used in Elderly Activity Life-Space Envelopes to describe node extent, with examples (nonexhaustive) from LSM literature.

10.2196/79308 Multimedia Appendix 3 Tables of ethnic distribution, educational level, and socioeconomic status of participants.

## Abbreviations

API: application programming interface
CMMSE: Chinese Mini-Mental State Examination
EASE: Elderly Activity Life-Space Envelopes
EQ-VAS: EuroQol Visual Analogue Scale
FMS: Future Mobility Survey
GIS: geographical information science
GIS: geographic information science
GPN: GPS node
LS: life spaces
LSM: life-space mobility
MDGA: multidomain geriatric assessment
MET: metabolic equivalent of task
NEWS: Neighborhood Environment Walkability Scale
NEWS-A: NEWS-Abbreviated
PTAL: Public Transport Accessibility Level
QoL: quality of life
SDN: self-declared node
UAB-LSA: University of Alabama at Birmingham’s Life Space Assessment
WHO-ICF: WHO International Classification of Functioning, Disability and Health
