# Supplementary material for: Elderly Activity Life-Space Envelopes (EASE): Development and Feasibility of a Comprehensive, Integrated Protocol for Life-Space Mobility Research in Population Health
Source: JMIR Res Protoc. 2025 Dec 19;14:e79308. doi: 10.2196/79308 (PMC12716834; doi:10.2196/79308)
Supplement: Multimedia Appendix 1 [file resprot-v14-e79308-s001.pdf]

LEARN MORE ABOUT MOBILITY!

VOLUNTEERS NEEDED
For a Community Project

Open to all 50 years old and above
Living in East, North-East, Central, and South Singapore

The SingHealth EASE Project helps you evaluate your health and risk of future disability, by exploring how and where you travel to meet your goals in life.

What is the EASE Project about?

The main aim is to measure Life Space, which is the area in which people move around in order to achieve self-care, employment, and other social and recreational needs.

What you will do:

- 60 - 90 Minutes
  Complete a questionnaire and some physical assessments
- Keep a travel log for 14 days
  (assisted by an app)

You will receive a physical assessment report and be reimbursed for your time

REGISTER NOW!

FOR MORE INFORMATION CONTACT US AT

Email: ease@singhealth.com.sg
Phone: 90357850

Organised by: SingHealth
In collaboration with: DukeNUS Medical School, Geriatric Education & Research Institute, SUTD, SIT, Mobile Market Monitor, People's Association, Our Tampines Hub, Whampoa, Pacific Activity Centre, Goodlife!, Presbyterian Community Services

了解您的社区活动能力！

征集社区项目志愿者

新加坡东部、东北部、中部和南部的所有50岁以上居民都可以参加

SingHealth EASE 项目通过探索您日常出行的方式和地点，从而帮助评估您的健康状况和未来罹患残疾的风险。

什么是EASE社区项目？

主要目的是衡量您的“生活空间”，即居民为实现自我保健、就业、社交和娱乐需求而进行的日常出行区域。

您将参与以下活动：

- **60 - 90** 分钟
  完成一份问卷，同时，工作人员将会对您进行健康状况评估
- 记录14天的出行记录
  （由手机应用程序协助完成）

您将收到一份健康状况评估报告。我们也将补偿您参与项目所花的时间。

现在就报名参加吧！

欲知更多项目信息，请联络我们：

电邮：**ease@singhealth.com.sg**
电话：**90357850**

项目主办机构： SingHealth
合作机构： DukeNUS Medical School, Geriatric Education & Research Institute, SUTD, SIT, Mobile Market Monitor, People's Association, Our Tampines Hub, Whampoa, Pacific Activity Centre, Goodlife!, Presbyterian Community Services

KETAHUI LEBIH LANJUT TENTANG MOBILITI!

SUKARELAWAN DIPERLUKAN
untuk Projek Kemasyarakatan

Terbuka kepada semua yang berusia 50 tahun ke atas
Menetap di daerah Timur, Timur Laut, Pertengahan/Bandar dan Selatan Singapura.

Projek EASE SingHealth dapat membantu anda untuk menilai kesihatan dan risiko kehilangan upaya pada masa hadapan anda, dengan meneroka bagaimana dan ke mana anda pergi untuk mencapai matlamat kehidupan anda.

Apakah itu Projek EASE?

Tujuan utamanya adalah untuk menilai Ruang Hidup, iaitu kawasan di mana seseorang berlegar untuk memenuhi penjagaan diri, pekerjaan, dan keperluan sosial serta rekreasi yang lain.

Apa yang anda akan lakukan:

- 60 - 90 minit
  Lengkapkan borang soal selidik dan jalani beberapa penilaian fizikal
- Simpan rekod perjalanan selama 14 hari
  (dengan bantuan aplikasi)

Anda akan menerima laporan penilaian fizikal dan pampasan untuk masa yang diluangkan

DAFTAR SEKARANG!

Untuk maklumat lanjut, hubungi kami di

Alamat e-mel: ease@singhealth.com.sg
Nombor Telefon: 90357850

Dianjurkan oleh: SingHealth
Dengan kerjasama: DukeNUS Medical School, Geriatric Education & Research Institute, SUTD, SIT, Mobile Market Monitor, People's Association, Our Tampines Hub, Whampoa, Pacific Activity Centre, Goodlife!, Presbyterian Community Services
