# Supplementary material for: Elderly Activity Life-Space Envelopes (EASE): Development and Feasibility of a Comprehensive, Integrated Protocol for Life-Space Mobility Research in Population Health
Source: JMIR Res Protoc. 2025 Dec 19;14:e79308. doi: 10.2196/79308 (PMC12716834; doi:10.2196/79308)
Supplement: Multimedia Appendix 2 [file resprot-v14-e79308-s002.pdf]

Measure	Description	Instrument reference and examples from LSM literature (non-exhaustive)
Minimal Convex Polygon (MCP) or Minimal Convex Hull (MCH) or Space Usage Level<br>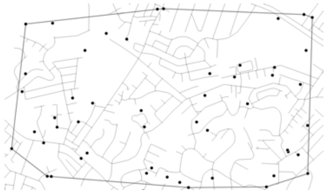	This spatial measure encompasses all GPS or node locations within the smallest possible polygon. This polygon is constructed by creating an envelope around all the GPS or node points recorded during a defined time period, with the outermost points serving as vertices. This area can be averaged daily over the study period to provide a measure of LSM. This is an 'unweighted' measure, and while the MCP captures the extreme extent of travel, it may include large geographic areas not actually visited by the individual.<br><br>Examples of mean sizes range from 52.9km^2 (Cologne, Germany [1]) and median sizes range from 7.73km^2 (Zurich, Switzerland [2]) to 17.5km^2 (Vancouver, Canada [3])	[1,3–10]
Standard Deviation Ellipse<br>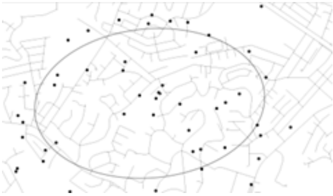	This is a spatial measure of the geographic concentration and dispersion of data points or nodes, providing a summary of the spatial distribution. It is defined by its center, orientation, and the lengths of its major and minor axes, which represent the spatial variability of the nodes. The major axis indicates the direction of maximum spread, while the minor axis shows the minimum spread. This 'weighted' method encompasses approximately 68% (i.e., one standard deviation) of the observed nodes, cantered on the mean center of these locations, and the ellipse's size is used to approximate the area within which a person moves over a specific period.<br><br>Examples of median sizes range from 2.43km^2 (New York, NY, USA [11]), 7.5km^2 (Gainesville, FL and New Haven, CT, USA [12]), 8.6km^2 (Hong Kong, China [13]), 11.2km^2 (Vancouver, Canada [3]) to 19.7km^2 (Detroit, MI, USA [14])	[1,3,10–14]

Road Network Buffer or Daily Path Area DPA<br>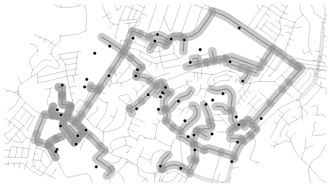	This spatial measure involves creating a buffer zone around road or path networks travelled by an individual.<br><br>An example is after using a 0.8 km buffer for all GPS points and dissolving into a single space, the median size was of 74.1sq^2 (Detroit, MI, USA [14]), Another example is that for a 1km buffer, the median size was 107km^2 (rural area, NC, USA [10])<br><br>The DPA may be a better measure of what the participant actually does compared to the MCH or SDE, however the time-space resolution of the GIS sensors needs to be high to obtain details. Also, calculations are complex with the significant amount of preparatory work with filtering and buffer algorithms from huge amounts of data [15,16].	[1,3,10,14]
Concave Hull<br>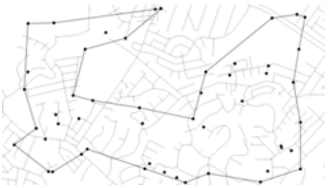	Unlike the convex hull, which forms the smallest convex polygon that can contain all the points, the concave hull provides a more flexible and accurate representation of the actual area covered by the points. It captures the true boundary of the points' distribution by excluding redundant areas and considering internal spatial heterogeneity. The concave hull is derived from algorithms like the Alpha shape algorithm, which helps in creating a more realistic boundary by including concave edges.	[17]
Radius of Gyration<br>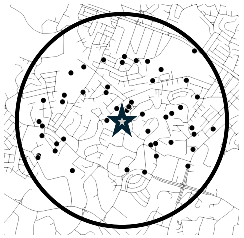	This is a spatial metric to measure the spatial dispersion of movement around a central point (e.g. home node). It is calculated with the formula:<br>$$r_g = \sqrt{\frac{1}{L}\sum_{i=1}^{L}(r_i - r_{cm})^2}$$<br>where:<br>• L is the number of stops made by the user over a given time frame.<br>• r_i the vector of coordinates of stop *i*.<br>• r_{cm} is the vector of coordinates of the center of mass, weighted by the duration of each stop Δt_i.	[4,6,18]

References

1. Giannouli E, Bock O, Mellone S, Zijlstra W. Mobility in old age: Capacity is not performance. BioMed Research International 2016;2016:1–8. doi: 10.1155/2016/3261567
2. Luo M, Kim E-K, Weibel R, Martin M, Röcke C. GPS-derived daily mobility and daily well-being in community-dwelling older adults. Gerontology 2023;69(7):875–887. doi: 10.1159/000527827
3. Hirsch JA, Winters M, Clarke P, McKay H. Generating GPS activity spaces that shed light upon the mobility habits of older adults: a descriptive analysis. Int J Health Geogr 2014 Dec;13(1):51. doi: 10.1186/1476-072X-13-51
4. Su R, Dodge S, Goulias KG. Understanding the impact of temporal scale on human movement analytics. J Geogr Syst 2022 July;24(3):353–388. doi: 10.1007/s10109-021-00370-6
5. Chung J, Boyle J, Wheeler DC. Relationship between life-space mobility and health characteristics in older adults using global positioning system watches. J Appl Gerontol 2022 Apr;41(4):1186–1195. doi: 10.1177/07334648211054834
6. Bayat S, Naglie G, Rapoport MJ, Stasiulis E, Widener MJ, Mihailidis A. A GPS-based framework for understanding outdoor mobility patterns of older adults with dementia: An exploratory study. Gerontology 2022;68(1):106–120. doi: 10.1159/000515391
7. Tung JY, Rose RV, Gammada E, Lam I, Roy EA, Black SE, Poupart P. Measuring life space in older adults with mild-to-moderate Alzheimer's Disease using mobile phone GPS. Gerontology 2014;60(2):154–162. doi: 10.1159/000355669
8. Luo N, Wang P, Thumboo J, Lim Y-W, Vrijhoef HJM. Valuation of EQ-5D-3L health states in Singapore: Modeling of time trade-off values for 80 empirically observed health states. PharmacoEconomics 2014 May;32(5):495–507. doi: 10.1007/s40273-014-0142-1
9. Ho Q, Lin W, Shaham E, Krishnaswamy S, Dang TA, Wang J, Zhongyan IC, She-Nash A. A distributed graph algorithm for discovering unique behavioral groups from large-scale telco data. Proceedings of the 25th ACM International on Conference on Information and Knowledge Management Indianapolis Indiana USA: ACM; 2016. p. 1353–1362. doi: 10.1145/2983323.2983354
10. Sherman JE, Spencer J, Preisser JS, Gesler WM, Arcury TA. A suite of methods for representing activity space in a healthcare accessibility study. Int J Health Geogr 2005 Dec;4(1):24. doi: 10.1186/1476-072X-4-24
11. York Cornwell E, Cagney KA. Aging in activity space: Results from smartphone-based GPS-tracking of urban seniors. The Journals of Gerontology: Series B 2017 Sept 1;72(5):864–875. doi: 10.1093/geronb/gbx063
12. Bai C, Zapata R, Karnati Y, Smail E, Hajduk AM, Gill TM, Ranka S, Manini TM, Mardini MT. Comparisons between GPS-based and self-reported life-space mobility in older adults. Health Informatics; 2022. doi: 10.1101/2022.09.19.22280116
13. Guo Y, Yeung C-Y, Chan GCH, Chang Q, Tsang HWH, Yip PSF. Mobility based on GPS trajectory data and interviews: A pilot study to understand the differences between lower- and higher-income older adults in Hong Kong. IJERPH 2022 May 3;19(9):5536. doi: 10.3390/ijerph19095536

14. Zenk SN, Schulz AJ, Matthews SA, Odoms-Young A, Wilbur J, Wegrzyn L, Gibbs K, Braunschweig C, Stokes C. Activity space environment and dietary and physical activity behaviors: A pilot study. Health & Place 2011 Sept;17(5):1150–1161. doi: 10.1016/j.healthplace.2011.05.001
15. Siła-Nowicka K, Vandrol J, Oshan T, Long JA, Demšar U, Fotheringham AS. Analysis of human mobility patterns from GPS trajectories and contextual information. International Journal of Geographical Information Science 2016 May 3;30(5):881–906. doi: 10.1080/13658816.2015.1100731
16. Long JA, Nelson TA. A review of quantitative methods for movement data. International Journal of Geographical Information Science 2013 Feb;27(2):292–318. doi: 10.1080/13658816.2012.682578
17. Bu J, Yin J, Yu Y, Zhan Y. Identifying the daily activity spaces of older adults living in a high-density urban area: A study using the smartphone-based Global Positioning System trajectory in Shanghai. Sustainability 2021 Apr 29;13(9):5003. doi: 10.3390/su13095003
18. Puthusseryppady V, Morrissey S, Aung MH, Coughlan G, Patel M, Hornberger M. Using GPS tracking to investigate outdoor navigation patterns in patients with Alzheimer Disease: Cross-sectional study. JMIR Aging 2022 Apr 21;5(2):e28222. doi: 10.2196/28222
