# Supplementary material for: Elderly Activity Life-Space Envelopes (EASE): Development and Feasibility of a Comprehensive, Integrated Protocol for Life-Space Mobility Research in Population Health
Source: JMIR Res Protoc. 2025 Dec 19;14:e79308. doi: 10.2196/79308 (PMC12716834; doi:10.2196/79308)
Supplement: Multimedia Appendix 3 [file resprot-v14-e79308-s003.pdf]

Table S1. Ethnic Distribution of Participants (N = 1118)

Ethnicity	Frequency	Percent
Chinese	1025	91.68
Malay	37	3.31
Indian	33	2.95
Others	23	2.06

Table S2. Highest Educational Level[1] of Participants (N = 1118)

Education	Frequency	Percent
No formal education	10	0.89
Primary	71	6.35
Secondary	336	30.05
Vocational / Institute of Technical Education (ITE)	45	4.03
Junior College (JC) / Polytechnic	260	23.26
University and above	396	35.42

Socioeconomic Status (SES) of Participants

As in many similar population health research studies, Socioeconomic Status (SES) is a sensitive and personal question, and participants were given the opportunity to decline answering if they were not comfortable. Non-response is a well-known challenge in this type of research.

To mitigate this potential data incompleteness and maximize usable data, the investigators asked about SES in two distinct ways: self-reported Monthly Household Income (Table S3) and self-reported Income Adequacy (Table S4). Participants were given the option to answer one, both, or decline entirely, providing a richer, multi-dimensional view of their economic standing.

Table S3. Self-reported Monthly Household Income (N = 1118)

Income	Frequency	Percent
Less than $1000	209	18.69
$1000–$3999	215	19.23
$4000–$6999	157	14.04
$7000–$9999	109	9.75
$10,000 and above	172	15.38
Declined to report	256	22.90

Table S4. Self-reported Income Adequacy (N = 1118)

Income Adequacy	Frequency	Percent
Much difficulty to meet expenses	26	2.33
Some difficulty to meet expenses	114	10.20
Just enough money, no difficulty	490	43.83
Enough money, with some left over	454	40.61
Declined to report	34	3.04

References

1. Ministry of Education. Overview of Singapore's Education System. Singapore: Ministry of Education; 2020. Available from: https://www.moe.gov.sg/
